# Computational and Experimental Investigation of Biofilm Disruption Dynamics Induced by High-Velocity Gas Jet Impingement

**DOI:** 10.1128/mBio.02813-19

**Published:** 2020-01-07

**Authors:** Lledó Prades, Stefania Fabbri, Antonio D. Dorado, Xavier Gamisans, Paul Stoodley, Cristian Picioreanu

**Affiliations:** a Department of Mining, Industrial and ICT Engineering, Universitat Politècnica de Catalunya, Manresa, Spain; b Perfectus Biomed Limited, Sci‐Tech Daresbury, Cheshire, United Kingdom; c Department of Microbial Infection and Immunity and Department of Orthopaedics, The Ohio State University, Columbus, Ohio, USA; d National Centre for Advanced Tribology at Southampton (nCATS), Department of Mechanical Engineering, University of Southampton, Southampton, United Kingdom; e Department of Biotechnology, Delft University of Technology, Delft, The Netherlands; Georgia Institute of Technology School of Biological Sciences

**Keywords:** non-Newtonian fluid flow, computational fluid dynamics, turbulence, high-velocity air jets, ripples, *Streptococcus mutans*, biofilms, disruption

## Abstract

Experimental data showed that high-speed microsprays can effectively disrupt biofilms on their support substratum, producing a variety of dynamic reactions such as elongation, displacement, ripple formation, and fluidization. However, the mechanics underlying the impact of high-speed turbulent flows on biofilm structure is complex under such extreme conditions, since direct measurements of viscosity at these high shear rates are not possible using dynamic testing instruments. Here, we used computational fluid dynamics simulations to assess the complex fluid interactions of ripple patterning produced by high-speed turbulent air jets impacting perpendicular to the surface of Streptococcus mutans biofilms, a dental pathogen causing caries, captured by high-speed imaging. The numerical model involved a two-phase flow of air over a non-Newtonian biofilm, whose viscosity as a function of shear rate was estimated using the Herschel-Bulkley model. The simulation suggested that inertial, shear, and interfacial tension forces governed biofilm disruption by the air jet. Additionally, the high shear rates generated by the jet impacts coupled with shear-thinning biofilm property resulted in rapid liquefaction (within milliseconds) of the biofilm, followed by surface instability and traveling waves from the impact site. Our findings suggest that rapid shear thinning under very high shear flows causes the biofilm to behave like a fluid and elasticity can be neglected. A parametric sensitivity study confirmed that both applied force intensity (i.e., high jet nozzle air velocity) and biofilm properties (i.e., low viscosity and low air-biofilm surface tension and thickness) intensify biofilm disruption by generating large interfacial instabilities.

## Abstract (precis)

Knowledge of mechanisms promoting disruption though mechanical forces is essential in optimizing biofilm control strategies which rely on fluid shear. Our results provide insight into how biofilm disruption dynamics is governed by applied forces and fluid properties, revealing a mechanism for ripple formation and fluid-biofilm mixing. These findings have important implications for the rational design of new biofilm cleaning strategies with fluid jets, such as determining optimal parameters (e.g., jet velocity and position) to remove the biofilm from a certain zone (e.g., in dental hygiene or debridement of surgical site infections) or using antimicrobial agents which could increase the interfacial area available for exchange, as well as causing internal mixing within the biofilm matrix, thus disrupting the localized microenvironment which is associated with antimicrobial tolerance. The developed model also has potential application in predicting drag and pressure drop caused by biofilms on bioreactor, pipeline, and ship hull surfaces.

## INTRODUCTION

During recent years, close attention has been paid to the mechanical behavior of biofilms when subjected to high shear turbulent flows, because of direct applications in utilizing such methods for biofilm removal. Fluid-biofilm patterns, such as formation of migratory ripples and surface instabilities, transition to fluid-like behavior, and stretching to finally break off the biofilm, have been described (1–5). Furthermore, the viscoelastic character of biofilms when exposed to turbulent flows has been reported (6–8), hypothesizing that generated instabilities enhance mass transfer into biofilms (9, 10).

Several biofilm “constitutive” models, mathematical models which describe the mechanical properties of material and how a material will respond to mechanical forces, have been developed with consideration of viscoelastic properties and biofilm-fluid interaction, as reviewed by different authors (11–14). Biofilm deformation and detachment under laminar flow conditions have been modeled using the phase field approach (15, 16) and the immersed boundary method (17). The movement of biofilm streamers (18) and the deformation of simple-shaped biofilm under high-speed flow (19) have been described using fluid-structure interaction models, implemented in commercial software packages (i.e., COMSOL Multiphysics and ANSYS). Small biofilm deformations have also been modeled considering the biofilm as a poroelastic material, compressing when exposed to laminar flows (20, 21). The effect of parallel air-water jets on the surface morphology of very viscous biofilms has been investigated numerically (3, 22), proposing the characterization of the biofilm rippling as Kelvin-Helmholtz instability. However, the interactions between biofilm and perpendicular impinging turbulent jet flow and how the turbulent impacts disrupt biofilm properties have not yet been described numerically. Therefore, a numerical investigation of the biofilm rippling patterns generated by turbulent jets could help clarify the mechanics behind the observed biofilm disruption. Computational fluid dynamics (CFD) is a useful tool for predicting the behavior of fluid flow and fluid-fluid interactions which might not be easily possible through experimentation. CFD models have been developed to describe air jet impingements into water vessels (23–25) using the volume of fluid (VOF) method to track the interface position between fluids. The VOF method has also been used to predict the wall shear stress produced by turbulent flows over biofilms (26) and to characterize biofilm removal by impinging water droplets (27). Nevertheless, both the turbulence effect on the growth surface and the biofilm as a distinct dynamic phase have not been considered. Thus, a CFD model for biofilm rippling under turbulent jets should include (i) multiphase flow with biofilm and air-water both as moving phases; (ii) accurate tracking of the biofilm-fluid interface with VOF; (iii) a reliable treatment of fluid dynamics at the biofilm growth surface (i.e., near-wall treatment) and at the biofilm-fluid interface (e.g., turbulent damping correction); and (iv) the biofilm phase as a non-Newtonian fluid, with liquefaction due to shear thinning at high shear rates.

The present study was aimed at developing a CFD model to characterize the observed dynamic rippling patterns of Streptococcus mutans biofilms exposed to high-velocity air jet perpendicular impingements. To this goal, two-dimensional (2D) axisymmetric CFD simulations were performed to describe the behavior of air-impinged biofilms, with consideration of turbulence and near-wall treatment. The non-Newtonian biofilm was examined under high shear rates to reveal the mechanisms driving its disruption under air jet impacts. The model was used to study the influence of parameters such as biofilm viscosity at rest and at predicted high shear rates, biofilm thickness, jet nozzle velocity, and air-biofilm surface tension on biofilm cohesiveness, deformation, and disruption.

## RESULTS

### Experimental results.

**FIG 1:**
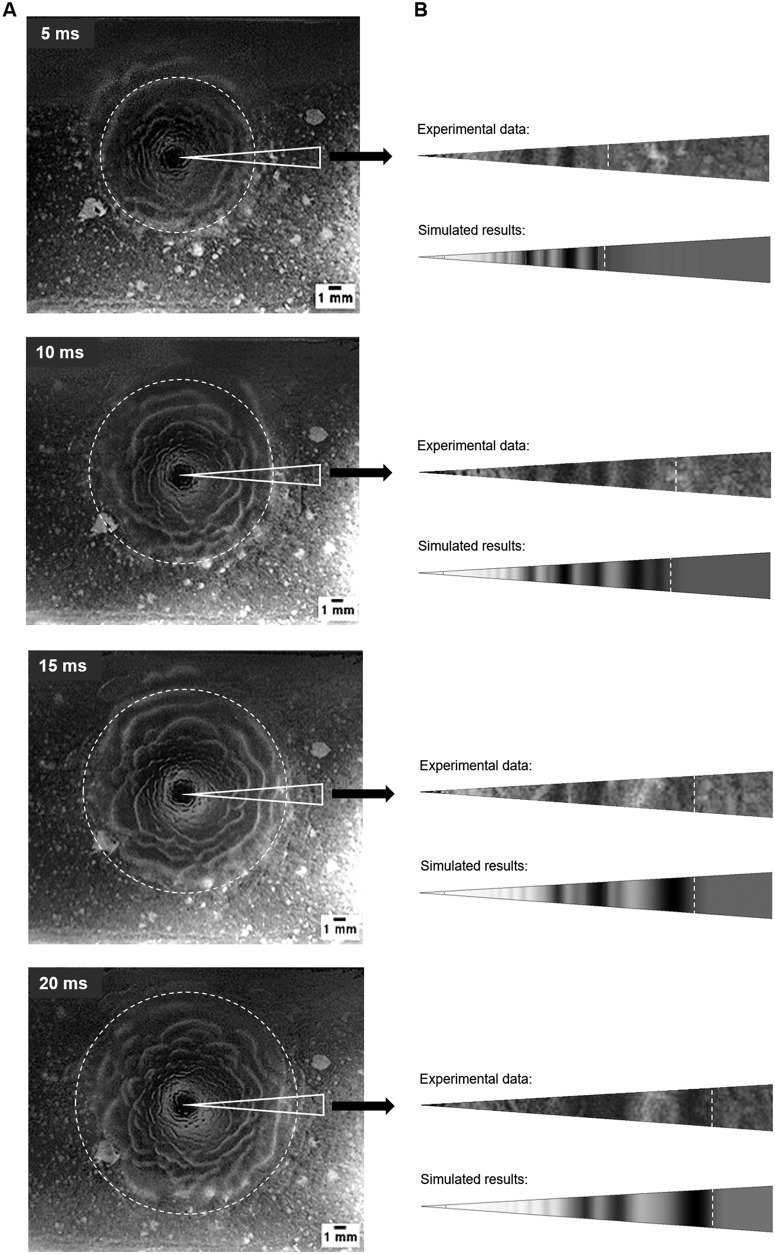
(A) Image frames at 5, 10, 15, and 20 ms from the high-speed movie. Thicker biofilm ripples and cell clusters outside the ripple zone are light, and the background slide surface is dark. (B) Comparison of measured and simulated biofilm displacement (right column) within the marked sector, with EVC_3_ and *γ* = 36 mN·m^−1^. The front position of the advancing ripples is indicated by the white dashed line. The gray scale in the simulations shows the local biofilm thickness, which is correlated with the wave amplitude. The high-speed recording of the jet impingement experiment is available in Video S1. The animation of the simulated ripple formation is presented in Video S2.

Figure 1A depicts image frames recorded at different times (5, 10, 15, and 20 ms) with a high-speed camera during perpendicular air jet impingement. The movies showed how the air jets first generated a clearing in the biofilm at the impingement site (which we refer to hereafter as a "cavity" in the biofilm), followed by the formation of surface instabilities which rapidly spread radially. The disrupted cavity area grew for approximately 200 ms until it stabilized with a diameter of approximately 1.5 cm. A movie showing the process is in Video S1 in the supplemental material. After ∼350 ms, the ripples died out when the biofilm had flowed to the cleared space edge (Fig. S2).

10.1128/mBio.02813-19.6 VIDEO S1 High-speed (2,000 frams per second [fps]) recording of the air jet impingement experiment on an S. mutans biofilm attached to a glass surface, at an air velocity of 41.7 m·s^−1^, with a nozzle diameter of 2 mm. The biofilm consisted or larger clusters (lighter patches) distributed heterogeneously and separated by a base biofilm (gray). In the first few frames, the shutter can be seen being lifted out of the way, exposing the biofilm to the air jet. Biofilm ripples are forming radially away from the central impact area. After approximately 200 ms, the biofilm had been “pushed” from the central area to from a cleared area (darker) of approximately 1.5 cm. This video was used to generate data for the computational analysis. Download Movie S1, AVI file, 13.0 MB. Copyright © 2020 Prades et al. 2020 Prades et al. This content is distributed under the terms of the Creative Commons Attribution 4.0 International license.

### Model verification with experimental data.

**(i) Biofilm viscosity assessments.** The shear-thinning character of S. mutans biofilms has been previously demonstrated by measuring the complex viscosity (28); however, no data are reported about their characterization as non-Newtonian fluids. The model development required a determination of the dynamic viscosity of S. mutans biofilms at much higher shear rates (10^4^ to 10^6^ s^−1^) than commonly reported for other biofilms (up to 2,000 s^−1^). Shear rate measurements in this range are impracticable with normal rheometric systems, which can achieve shear rates of around 10^4^ s^−1^. Microchannels containing microelectromechanical systems (MEMS) pressure sensors have been used to achieve a rate of almost 10^5^ s^−1^ (29); however, even this is still an order of magnitude less than the predicted shear rate experienced by the biofilm in our experiments. Thus, we were forced to extrapolate the values used in the model from existing data. The complex viscosity can be related to the dynamic viscosity by the empirical Cox-Merz rule (30) (i.e., the two viscosity measures should be identical at comparable observation time scales). However, some discrepancies in the Cox-Merz rule have been reported in samples with gel characteristics, such as polysaccharides, and biofilms by obtaining larger values for the complex viscosities (31–33), probably due to physical and chemical interactions present in these samples (34).

**FIG 2:**
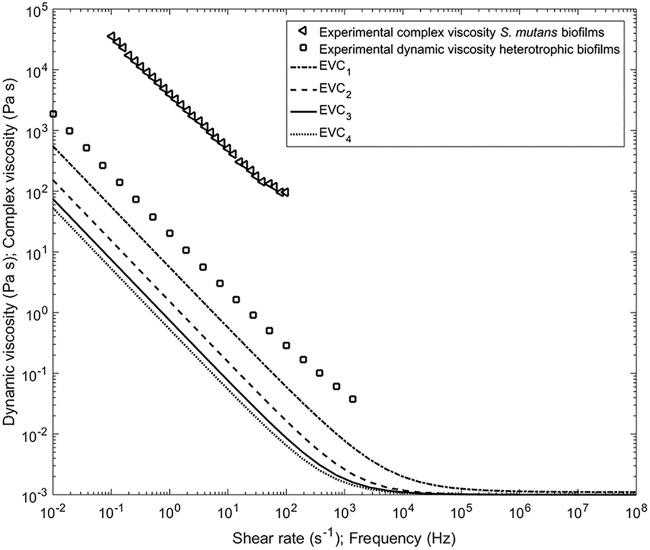
Dependency of biofilm viscosity on shear rate. Shown is the experimental dynamic viscosity for the heterotrophic biofilm (squares) function of shear rate (31) and the complex viscosity of the S. mutans (triangles) as a function of frequency (28). Solid lines represent estimated viscosity curves (EVC) with different parameter values as shown in Table 1.

Assuming that the dynamic viscosity should be lower than the complex viscosity, parametric sweeps were performed to evaluate the dynamic viscosity model (Equation 3). As an example, four estimated viscosity curves (EVC_1_, EVC_2_, EVC_3_, and EVC_4_) were represented together with the experimental reference values in Fig. 2. EVC_1_ corresponded to the highest dynamic viscosity, while EVC_4_ was the lowest viscosity curve. To reproduce the observed liquefaction behavior (9, 49), the Herschel-Bulkley curves were adjusted to bend asymptotically to that of water viscosity at very high shear rates, with the reasoning that water represents the lowest possible limit for a completely broken-down hydrogel. However, in reality, the biofilm viscosity is expected to be higher than that of water since even if completely mixed will contain cells and expolysaccharide (EPS) components. The Herschel-Bulkley parameters and the shear rate thresholds at which biofilm viscosity reached the viscosity of water are listed in Table 1.

**TABLE 1:** Rheological parameters of Herschel-Bulkley model and shear rate thresholds at which the biofilm viscosity reached the water viscosity for the four estimated viscosity curves (EVC)

EVC	Data for parameter^*a*^:			
	*σ_y_* (Pa)	*K* (Pa⋅s)	*n* (-)	$\dot{\gamma }$_w_ (s^−1^)
EVC_1_	5.529	0.0407	0.477	>10^8^
EVC_2_	1.529	0.0012	0.568	10^6^
EVC_3_	0.745	0.0010	0.600	2 × 10^6^
EVC_4_	0.529	0.0013	0.550	2 × 10^5^

a *σ_y_*, yield stress; *K*, fluid consistency index; *n*, flow behavior index; $\dot{\gamma }$_w_, shear rate threshold at which biofilm viscosity reduced to that of water.

**FIG 3:**
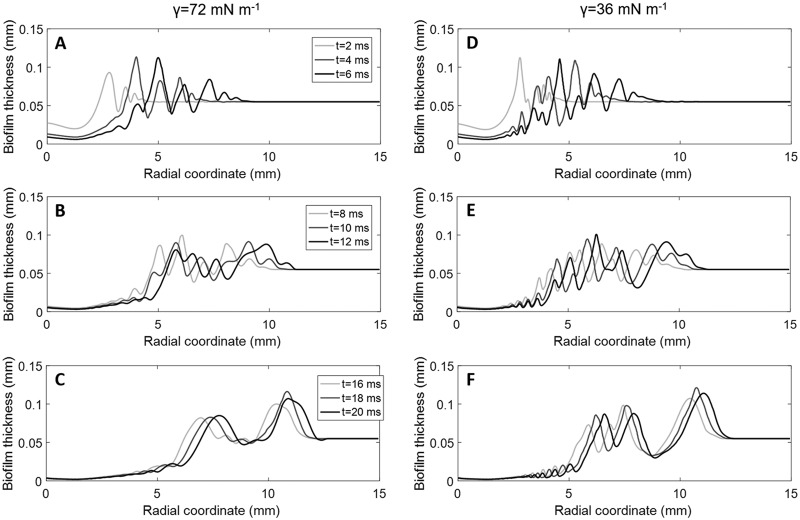
Simulated changes in biofilm thickness in time as a function of radial distance from the point of impingement (from 2 to 20 ms) for the viscosity model EVC_3_ and two surface tensions, (A to C) *γ* = 72 mN·m^−1^ and (D to F) *γ* = 36 mN·m^−1^. An animation of the simulated biofilm rippling can be found in Video S3.

**(ii) Computed results.** For the experimental air inlet velocity (*u_j_ *= 41.7 m·s^−1^), the biofilm response was simulated with different viscosity curves (Table 1) and two surface tension values (*γ*) of the air-biofilm interface (*γ* = 72 mN·m^−1^ and *γ* = 36 mN·m^−1^). The former is for air-water *γ* at 20°C, considering the biofilm matrix to be more than 90% water (35), and the latter smaller value is from Koza et al. (36) since the amphiphilic nature of EPS can have a surface-active effect (37), altering the air-water *γ*. The image frames taken during the biofilm disruption at different times were compared with the simulation results. The estimated viscosity curve EVC_3_ with *γ* = 36 mN·m^−1^ best matched the experimental data, as illustrated in Fig. 1B, with respect to the distance reached by the traveling wave front and the position of several ripple maximal and minimal thicknesses (dark and light areas in the simulation results, respectively). An animation of the simulated ripple formation is presented in Video S2. Figure 3 depicts the biofilm surface contours over time for cases simulated with EVC_3_ and *γ* = 72 mN·m^−1^ (left) or *γ* = 36 mN·m^−1^ (right) from the early cavity formation (Fig. 3A and D) until the deformation of wave damping (Fig. 3C and F). An animation of the simulated biofilm rippling can be found in Video S3. A lower surface tension clearly intensified the disruption and formation of surface instabilities, which were qualitatively analyzed at 4 and 20 ms (Fig. 3). Ripples began to form from 3 and 5 mm at 4 and 20 ms, respectively. At early times (4 ms), the cavity width was ∼2 mm, and the depth reached >80% of the initial biofilm thickness. Later, at 20 ms, the cavity width extended to ∼4 mm, and the depth reached almost to the biofilm substratum, creating a zone cleared of biofilm with a radius of about 2 mm. The disruption caused a biofilm deceleration of ∼0.025 m·s^−2^.

10.1128/mBio.02813-19.7 VIDEO S2 Simulated biofilm ripple formation in time (*x*-*y* top view), from a biofilm thickness of 55 μm with viscosity model EVC3 and surface tension γ = 36 mN·m^−1^, subjected to a perpendicular air jet with velocity of 41.7 m·s^−1^. Download Movie S2, AVI file, 12.1 MB. Copyright © 2020 Prades et al. 2020 Prades et al. This content is distributed under the terms of the Creative Commons Attribution 4.0 International license.

10.1128/mBio.02813-19.8 VIDEO S3 Simulated changes of biofilm thickness in time (0 to 20 ms) over the radial direction (*x*-*z* lateral view) for viscosity model parameter EVC_3_ and surface tension *γ* = 36 mN·m^−1^. Download Movie S3, AVI file, 14.6 MB. Copyright © 2020 Prades et al. 2020 Prades et al. This content is distributed under the terms of the Creative Commons Attribution 4.0 International license.

**FIG 4:**
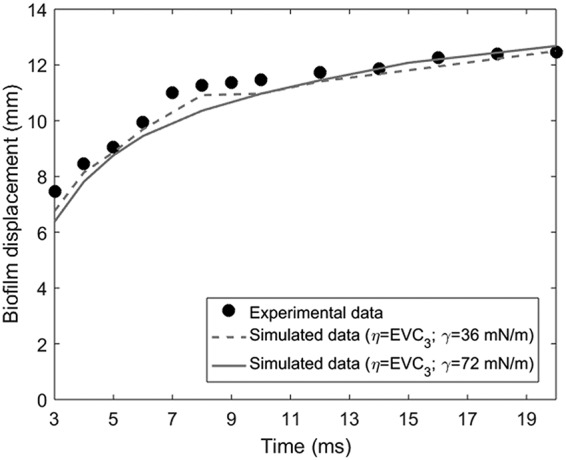
Experimental (symbols) and simulated data (lines) of biofilm displacement as a function of jet exposure time. Parameter values for each of the simulation runs are indicated in the legend.

By tracking the position of the advancing front of the ripples, the biofilm displacement was determined as defined as the maximum distance traveled by the advancing front of the ripples over the underlying biofilm support at a given time (5). From the movies, an average displacement was computed from the front positions in eight radial directions at each time (Fig. 1), whereas the biofilm displacement in the simulations was computed in one radial direction because of the axial symmetry of the computational domain. Figure 4 compares the experimental and simulated displacements, where biofilm responses were computed with EVC_3_ and two surface tensions. Initially, the disruption front moved quickly but slowed down and reached a steady value after about 20 ms, trends reflected in both the experiments and model. It seems that the lower surface tension allowed for a faster displacement (i.e., less opposing force to the air stream) and better fit of the experimental data, but the differences were still too small to be considered significant.

**FIG 5:**
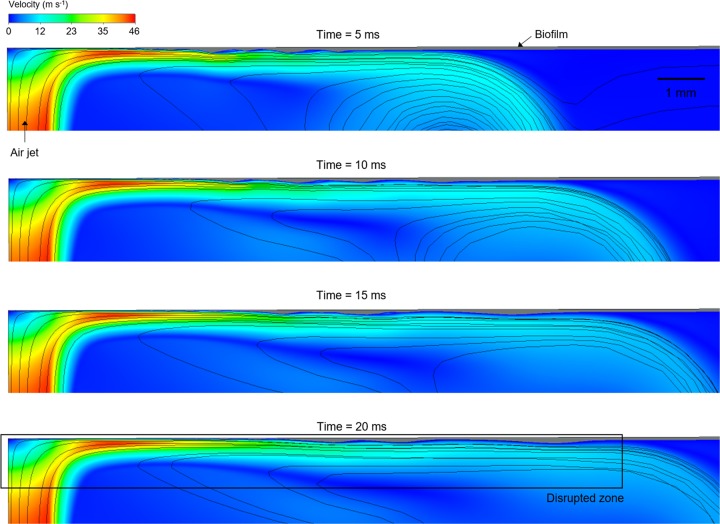
Simulated sequence (5, 10, 15m and 20 ms) of the air jet impingement over the biofilm for *η* = EVC_3_ and *γ* = 36 mN·m^−1^. The velocity magnitude of the airflow is represented by the colored surface, while the biofilm is the gray area on the top side. Air streamlines and flow directions are also displayed. (For an interpretation of the references to color in this figure, see the Web version of this article.) An animation of the simulated biofilm rippling can be found in Video S4.

A simulated sequence of the air jet impingement over the biofilm in the lateral view showing fluid-biofilm interaction is presented in Fig. 5 (for an animation, see Video S4). Initially, the airflow flowed faster than did the biofilm, forming ripples on the biofilm surface until a steady state was reached after 20 ms, generating a disrupted zone with a ∼13 mm radius. The velocity field shows the high velocity around the air nozzle and continuously decreasing velocities as the air flows radially along the biofilm surface far from the impact zone. The airflow loses its kinetic energy as it expands radially, and at a certain distance from the center, the shear would be too weak to deform the biofilm any further; therefore, a steady state was reached.

10.1128/mBio.02813-19.9 VIDEO S4 Simulated biofilm ripple formation over time (*x*-*z* lateral view), from a biofilm thickness of 55 μm with viscosity model EVC_3_ and surface tension *γ* = 36 mN·m^−1^, subjected to a perpendicular air jet with velocity of 41.7 m·s^−1^. Colored surface, velocity magnitude (meters per second); gray surface, biofilm area. In this simulation, the jet is coming from the bottom of the screen, and the biofilm is on the top. Download Movie S4, AVI file, 2.3 MB. Copyright © 2020 Prades et al. 2020 Prades et al. This content is distributed under the terms of the Creative Commons Attribution 4.0 International license.

**FIG 6:**
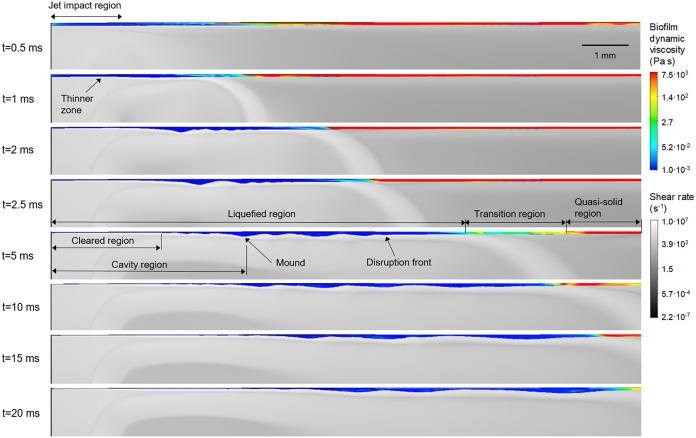
Simulated distributions of biofilm dynamic viscosity (color scale area) and shear rate (gray scale area) in the biofilm-disrupted region at different times (*η* = EVC_3_, *γ* = 36 mN·m^−1^). Both biofilm viscosity and shear rate are displayed on logarithmic scales. (For an interpretation of the references to color in this figure, see the Web version of this article.) An animation of the simulated biofilm rippling can be found in Video S5.

A sequence of the air shear rate and the biofilm dynamic viscosity distributions is presented in Fig. 6 (for an animation, see Video S5), and the pressure and velocity component profiles are depicted in Fig. S3 and S4, respectively. The simulations showed high shear rates (∼10^6^ to 10^7^ s^−1^), which directly produced high shear stresses (∼10^4^ Pa) and relative pressure (∼1,400 Pa) in the air jet impact zone during air jet exposure. Significantly, the high shear rates generated on the air-biofilm interface rapidly reduced the biofilm dynamic viscosity from the initial value (7,500 Pa⋅s) to that of water (0.001 Pa⋅s), suggesting that complete liquefaction of the biofilm had occurred.

10.1128/mBio.02813-19.10 VIDEO S5 Simulated biofilm ripple formation over time (*x*-*z* lateral view), from a biofilm thickness of 55 μm with viscosity model parameter EVC_3_ and surface tension *γ* = 36 mN·m^−1^, subjected to a perpendicular air jet with velocity of 41.7 m·s^−1^. Gray-scale surface, shear rate (per second); color scale, biofilm dynamic viscosity (Pa⋅s). In this simulation, the jet is coming from the bottom of the screen, and the biofilm is on the top. Download Movie S5, AVI file, 4.3 MB. Copyright © 2020 Prades et al. 2020 Prades et al. This content is distributed under the terms of the Creative Commons Attribution 4.0 International license.

10.1128/mBio.02813-19.3 FIG S2 Steady state of the biofilm disruption after perpendicular air jet impingement. The ripples die out when the biofilm flowed to the cleared space edge after ∼350 ms of jet exposure. Download FIG S2, TIF file, 0.2 MB. Copyright © 2020 Prades et al. 2020 Prades et al. This content is distributed under the terms of the Creative Commons Attribution 4.0 International license.

10.1128/mBio.02813-19.4 FIG S3 Computed pressure fields in the disrupted region (rectangle marked in Fig. 5) for different times, i.e., 0.7, 0.8, 0.9, 1, 2, 2.5, 5, 10, 15, and 20 ms. Simulations were performed with *η* = EVC_3_ and *γ* = 36 mN·m^−1^. Color scale shows pressure in pascals. Larger pressures were in the air jet impact zone. Pressure gradients formed in the in the biofilm phase were observed from *t* = 2 ms. Download FIG S3, TIF file, 0.4 MB. Copyright © 2020 Prades et al. 2020 Prades et al. This content is distributed under the terms of the Creative Commons Attribution 4.0 International license.

10.1128/mBio.02813-19.5 FIG S4 Computed air jet velocity profiles in *x* and *y* directions (velocity *u_r_* and *u_z_*, respectively) in the disrupted region (see Fig. 5) for different times, i.e., 0.7, 0.8, 0.9, 1, 2, 2.5, 5, 10, 15, and 20 ms. Simulations were performed with *η* = EVC_3_ and *γ* = 36 mN·m^−1^. Color scale shows velocity in meters per second. Larger velocities in the gas phase were produced in the tangential direction to the biofilm phase, reaching maximum values of 45 m·s^−1^. Download FIG S4, TIF file, 1.2 MB. Copyright © 2020 Prades et al. 2020 Prades et al. This content is distributed under the terms of the Creative Commons Attribution 4.0 International license.

The velocity component in the radial direction (*u_r_*, parallel with the initial biofilm surface) was dominant over the component in the axial direction (*u_z_*), which indicated the drag direction on the biofilm surface (Fig. S4). These shear forces and the pressure produced changes in biofilm thickness beginning from ∼0.7 ms in the impact zone. A slightly uneven biofilm surface can be observed in the area disrupted by the jet (Fig. 6 and S3); thus, both pressure and shear stress forces initiate biofilm movement. Between 1 and 2 ms, the first biofilm ripples started to form. The ripple formation coincided with a larger biofilm area being liquefied, from a radius of 5 mm liquefied at 2 ms to about 8 mm at 5 ms. Interestingly, the movement of this part of the fluidized biofilm produced pressure oscillations within the biofilm (Fig. S3), thus generating the ripples. The largest gradients of pressure were observed for 5 ms and decreased further when the ripples were near a steady state (from 15 ms). To characterize the biofilm ripples, the wavelength, characteristic frequency, and ripple velocity were determined for both experimental and simulated data (*η* = EVC_3_, *γ* = 72 and 36 mN·m^−1^). The values averaged in time are listed in Table 2, showing good agreement between the simulated and experimental results.

**TABLE 2:** Ripple characterization by average wavelength, frequency, and average velocity

Group	Mean ± SD^*a*^		
	λ_R_ (mm)	f (Hz)	u_R_ (mm⋅s^−1^)
Experimental	0.9 ± 0.3	367	330 ± 110
Simulated (*η* = EVC_3_, *γ* = 72 mN/m)	1.012 ± 0.13	311	315 ± 40
Simulated (*η* = EVC_3_, *γ* = 36 mN/m)	1.047 ± 0.14	383	401 ± 54

a λ_R_, average wavelength; f, frequency; u_R_, average ripple velocity.

### Sensitivity analysis.

**FIG 7:**
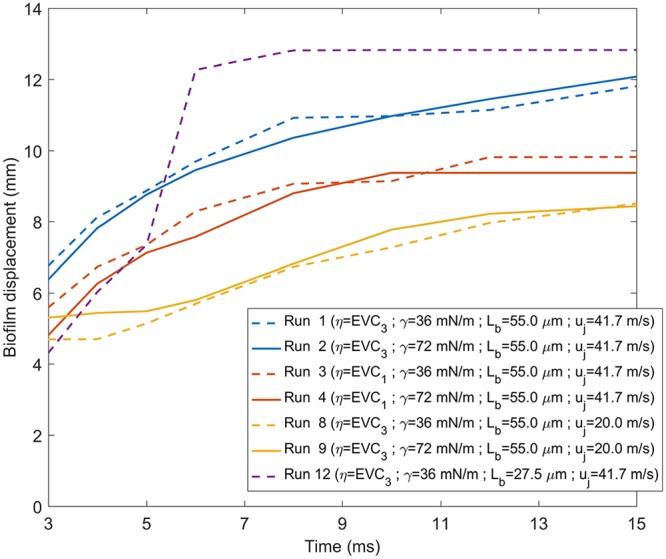
Parametric study of the biofilm displacement (in millimeters) over jet exposure time (in milliseconds) for different model parameters, as follows: biofilm viscosity *η* (runs 1 and 2 versus runs 3 and 4), jet velocity *u_j_* (runs 1 and 2 versus runs 8 and 9), and biofilm thickness *L_b_* (run 1 versus run 12). Solid lines indicate the simulations computed with surface tension *γ* = 72 mN·m^−1^, and dashed lines indicate the simulations computed with surface tension *γ* = 36 mN·m^−1^. Parameter values for each of the runs are indicated in the legend. (For an interpretation of the references to color in this figure, see the Web version of this article.)

**FIG 8:**
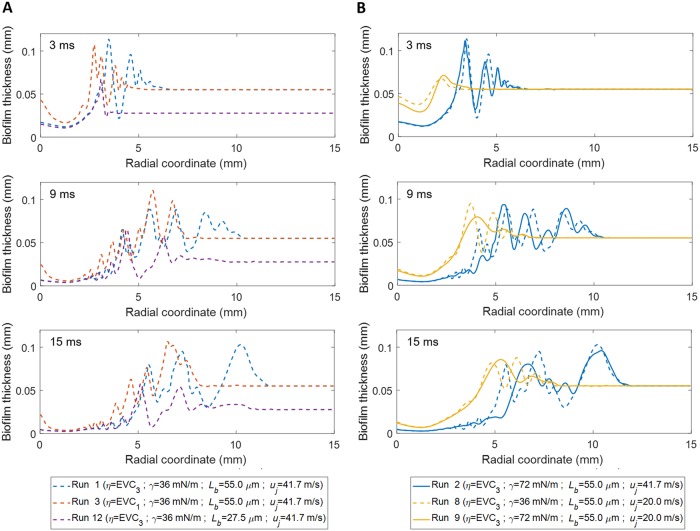
Sensitivity analysis data for the biofilm disruption produced by air jet impingement for different model parameters are as follows: (A) biofilm viscosity functions EVC (run 1 versus run 3) and biofilm thickness *L_b_* (run 1 versus run 12) and (B) jet velocities *u_j_* (run 1 versus 8 and run 2 versus 9). Parameter values for each of the runs are indicated in the legend. (For interpretation of the references to color in this figure legend, see the Web version of this article.)

A sensitivity analysis was performed to determine the implications of the different model parameters on the biofilm disruption strategies, analyzing the biofilm displacement (Fig. 7) and the development of the biofilm-cleared zone and the surface instabilities (Fig. 8), both over jet exposure time. Parametric simulations were performed with changes in air velocity, biofilm thickness, biofilm viscosity, and air-biofilm surface tension.

**(i) Biofilm viscosity (*η*).** Biofilms with higher viscosity (*η* = EVC_1_, runs 3 and 4) underwent smaller biofilm displacement due to the higher resistance to flow (Fig. 7). Values below *η* = EVC_3_ (runs 1 and 2) meant a softer structure, disrupting the full biofilm length after only a few milliseconds of jet exposure, while experimentally, the biofilm displacement was less than 6 mm for 2 ms. The biofilm with the lowest viscosity (*η* = EVC_3_, run 1) was disrupted over the largest radius, while biofilm residues remained unremoved in the cavity center for the more viscous biofilm (*η* = EVC_1_, run 3) (Fig. 8). Thus, expectedly, low values of biofilm viscosity lead to greater biofilm displacement and removal.

**(ii) Surface tension (*γ*).** A lower surface tension (*γ* = 36 mN·m^−1^, runs 1 and 3) allowed for quicker biofilm displacement initially (Fig. 7) than with *γ* = 72 mN·m^−1^ (runs 2 and 4), until arriving at similar steady-state displacement, possibly explained by the stabilization effect of surface tension. Moreover, the lower surface tension produced ripples with higher frequency and higher amplitude, i.e., the biofilm surface was less unstable (Fig. 8). However, the cavity depth was not affected by the surface tension in the range of the analyzed values.

**(iii) Jet velocity (*u_j_*).** The largest and fastest displacements were achieved with high velocity (runs 1 and 3) due to the higher shear rates produced (Fig. 7). For low velocity (runs 8 and 9), the biofilm started to move 5 ms later than for high velocity because the slower air jet reached the biofilm with the corresponding delay, generating also a smaller biofilm cavity (Fig. 8B). Additionally, at *u_j_* = 60 m·s^−1^, the biofilm was removed much faster with full-length disruption after just 2 ms. In general, as the gas jet velocity increased, the central biofilm cavity got deeper and wider, with the rim of the rising above the original biofilm level, while the biofilm surface became more unstable, suffering larger surface perturbations.

**(iv) Biofilm thickness (*L_b_*).** The thinner biofilm (*L_b_* = 27.5 μm, run 12) was moved faster by the air jet and, consequently, reached stationary state sooner, i.e., in less than 8 ms, compared with >20 ms for the thicker film (*L_b_* = 55 μm, run 1) (Fig. 7). The thinner biofilm appeared to be slightly more stable than the thicker biofilm, displaying fewer ripples (Fig. 8A) and reaching steady-state more quickly, possibly due to having less biomass to displace. In addition, the cavity shapes were very similar for the different biofilm thicknesses analyzed.

## DISCUSSION

### Disruption dynamics.

Three distinct phases were identified during biofilm disruption by analyzing the development of the biofilm ripple patterns. In the first phase from 1 to 6 ms (Fig. 3A and D), the ripples had a relatively regular wavelength and amplitude for the first wave formed, followed by a series of smaller waves until total wave decay at the disruption front. In the second phase from 7 to 14 ms (Fig. 3B and E), the waves appeared distorted, with a reduced amplitude and a more constant wavelength over the disruption area (i.e., the initial, smaller waves on the tail grow larger). Finally, from 15 to 20 ms (Fig. 3C and F), there were fewer ripples but with larger wavelengths than in the previous phases. Particularly, such wavelengths and ripple velocity characterizing perpendicular impingements (Table 2) were similar to those measured on S. mutans bacteria exposed to air jets applied parallel to the surface (3).

The ripple dynamics produced by turbulent flow over biofilms has been previously related to the viscoelastic nature of biofilms (5, 7, 38), suggesting that the biofilm mechanical response is dominated by the EPS matrix properties (6, 39). Klapper et al. (6) hypothesized that the EPS matrix responds to stress by exhibiting first an elastic tension caused by the combination of polymer entanglement and weak hydrogen bonding forces; second, it exhibits a viscous damping, where energy is absorbed as the biofilm flows and acts like a shock absorber due to the polymeric friction and hydrogen bond breakage; and third, it exhibits polymer alignment in the shear direction, possibly leading to a shear-thinning effect in which the viscosity is lowered as the polymeric network structure of the biofilm matrix breaks down. The elastic tension may be related to the first disruption phase, where waves with similar patterns were generated in response to the initial jet impingement. The viscous damping could correspond to the second disruption phase with distorted ripples. The polymer alignment could be associated with the last phase, where the ripples practically stop moving and reach steady state. Possibly, the wave decay (where the ripples died down) occurred because the energy transmitted from the air to the biofilm at this distance and time were less than the viscous dissipation in the biofilm (40), being balanced by biofilm internal cohesive forces.

Interestingly, biofilm displacement reached a quasi-steady state as the air jet velocity also approached steady state. This suggests that the relaxation time of airflow was on the same order as the plastic relaxation time of the biofilm. However, the relatively slow continuation of the biofilm movement after the airflow reached the quasi-stationary regime indicated the continuation of viscous damping within the biofilm.

Some model assumptions may compromise the accuracy of the determined ripple patterns. Assuming constant properties such as thickness and viscosity on the biofilm model has an effect on the ripple pattern formation. Further experimental techniques should be used to reveal heterogeneity in biofilm properties and to include them in future simulations. In addition, an accurate evaluation of the biofilm disruption should be done with three-dimensional (3D) models. The difficulty, however, is not only the definition of 3D geometry and properties into the computational model but, more significantly, a large increase in computing time.

### Conditions promoting disruption.

Our model suggests that the air jet exposures generating high shear rates, coupled with the biofilm shear-thinning behavior, produced rapid (within milliseconds) biofilm disruption, thus affecting both the biofilm properties and the applied force intensity. The greatest interfacial instabilities are produced by the largest forces (i.e., high air jet velocity) and the lowest values of biofilm properties (i.e., low viscosity and air-biofilm surface tension and thickness).

The similarity between the modeling and the experimental measurements suggests non-Newtonian fluid behavior of S. mutans biofilms. Biofilm liquefaction, i.e., the complete breakdown of polymer network interactions in the biofilm matrix, is a mechanism that can explain the extremely quick disruptive effect induced by high shear airflows on the biofilms. The Herschel-Bulkley parameters of the estimated biofilm viscosity curve EVC_3_ described the required shear-thinning behavior, with the fitted yield stress (*σ_y_* = 0.745 Pa) in accordance with the viscoelastic linearity limit (*σ* = 3.5 Pa) previously determined by creep analysis (28). Biofilms in general show mechanically viscoelastic behavior (38, 39); however, the consistent results obtained considering the biofilm as a non-Newtonian fluid indicate that under turbulent flows, the biofilms elastic behavior can be neglected, as recently reported (22). Additionally, biofilm expansion under noncontact brushing is attributed to its viscoelastic nature (41). Here, there was no evidence that the biofilm structure was expanded during impingement. Finally, although biofilm grown from a single species was analyzed here, the literature shows remarkable similarity in the viscoelastic responses of many different types of biofilms when subject to shear stresses, even though the magnitude of the elastic and viscous moduli vary over many orders of magnitude (42); thus, it is reasonable to conclude that biofilms formed from other species might exhibit flow behavior similar to that described here, as suggested by the ripple patterns experimentally observed in Pseudomonas aeruginosa and Staphylococcus epidermidis biofilms exposed to high-velocity shear flows (3).

Furthermore, the observed results highlight the importance of considering the correct representation of forces which mechanically can disrupt biofilms. The numerical simulations indicated that inertial and interfacial tension forces are governing biofilm disruption by impacting turbulent air jets, as seen with the fluid dynamic activity reported for microdroplet sprays and power toothbrushes (27, 41). Specifically, the size and geometry of the cavity formation depends on a force balance at the free surface (23), assuming in our case that inertial forces determine the cavity depth, while the width was determined by both inertia and surface tension, evidenced by the presence of small-amplitude ripples at the cavity edge. Ripples were produced due to pressure and shear stress variations in the gas transmitted to the wavy surface (40). For very thin fluid films, the fluctuations in the fluid have much larger components in the tangential direction than in the normal direction; consequently, the shear stress is the dominant mechanism (43), as shown in our case, in which a significant role for pressure variation was also identified. Therefore, the following two mechanisms were determined to produce moving biofilm ripples as a result of air jet impingement: (i) pressure oscillations generate biofilm ripples, and (ii) friction forces drag the biofilm along the support surface. Last, the simulation also revealed the important role of interfacial tension forces in the formation of surface instabilities, with less surface tension leading to more rippling (i.e., higher frequency and velocity). These results are in agreement with the possibly lower surface tension for biofilm-air (*γ* = 36 mN·m^−1^) than for water-air (*γ* = 72 mN·m^−1^), as measurements for Bacillus subtilis, Pseudomonas fluorescens, and P. aeruginosa biofilms indicated values within the range of 25 to 50 mN·m^−1^ (36, 44, 45). The amphiphilic character and surfactant production are associated as main effects controlling surface tension in microbial colonies and biofilms (45, 46), being attributed the surface tension reduction due to the presence of surfactants (36, 39, 45). Biofilm surface tension differences could also explain the different ripple patterns observed between S. mutans biofilms and biofilms grown from Pseudomonas aeruginosa and Staphylococcus epidermidis (3). A lower surface tension intensified the formation of small-amplitude waves near the impact zone (i.e., the more “flexible” interface was more wrinkled). This increase in air-biofilm interfacial area could enhance the friction to flow. Moreover, there could be implications on the mass transfer, in that wavy interfaces will distort the diffusion boundary layer, and interfacial waves have been related to mass transfer enhancement (47, 48).

These results contribute to the existing knowledge of mechanisms promoting disruption though mechanical forces, opening new ways to optimize biofilm control strategies which rely on fluid shear. The model can be used to determine optimal parameters (e.g., jet velocity and position and angle of attack) to remove or predict the spread of the biofilm in specific applications (e.g., in dental hygiene or debridement of surgical site infections). The developed model also has potential application in predicting drag and pressure drop caused by biofilms in bioreactors, industrial pipelines, and ship hull surfaces, as well as predicting how shear might influence the removal or spread of biofilms associated with medical devices such as orthopedic implants, voice prostheses, catheters, and vascular stents.

## MATERIALS AND METHODS

### Biofilm growth.

S. mutans UA159 (ATCC 700610) biofilms were grown for 72 h on glass microscope slides at 37°C and 5% CO_2_ in brain heart infusion (Sigma-Aldrich, St. Louis, MO) supplemented with 2% (wt/vol) sucrose (Sigma-Aldrich) and 1% (wt/vol) porcine gastric mucin (type II; Sigma-Aldrich). After the growth period, the biofilm-covered slides were gently rinsed in 1% (wt/vol) phosphate-buffered saline solution (Sigma-Aldrich) and placed in petri dishes (49). Biofilm thickness was determined by fixing untreated samples with 4% (wt/vol) paraformaldehyde and staining with Syto 63 (Thermo Fisher Scientific, UK). Subsequently, three random confocal images were taken on three independent replicate biofilm slides, with a thickness of 51.8 ± 4.9 μm measured using COMSTAT software; thus, we used a biofilm thickness (*L_b_*) of 55 μm in the simulations.

### Biofilm perpendicular air jet impingement.

An air jet generated from a piston compressor (ClassicAir 255; Metabo, Nürtingen, Germany) impinged on biofilm samples at a 90° angle. Experiments were performed in triplicate. The compressor tip (internal diameter, 2 mm) was held at a 5 mm distance from the biofilm. The air jet impingement was recorded at 2,000 frames per second with a high-speed camera (MotionPro X3; IDT Vision, Pasadena, CA) placed to record the back view of the biofilm-covered microscope slide. The average air jet velocity exiting the nozzle (*u_j_* = 41.7 ± 1.5 m·s^−1^) was measured with a variable area flow meter. To estimate the Reynolds number for the biofilm (Re_b_) flowing along the substratum, the biofilm thickness (*L_b_*) was used as the characteristic length, and the biofilm density (*ρ_b_*) was assumed to be equal to that of water. The variation of biofilm displacement velocity (*u_b_*) and biofilm viscosity (*η*) variables had greater effect in the Re_b_. At the highest shear stresses, the biofilm behaved as if it was completely liquefied to water, with *η* = 0.001 Pa⋅s (see Results and Fig. 2), and moved with a maximum velocity *u_b_* ≈ 0.2 m⋅s^−1^. Under these conditions, Re_b_ = 11 indicates laminar flow. Considering the viscosity and density of air at 20°C and 1 atm, and the characteristic length equal to the nozzle tip diameter, an estimated maximum Reynolds number of the air jet (Re_a_) was 5,600, which is in the turbulent regime (50).

### Data postprocessing.

Fast Fourier transform (FFT) was used to determine the dominant period (T) and dominant frequency (f) of the ripple patterns in the S. mutans biofilm formed during exposure to the air jet. Frames from the experimental high-speed video and data exported from the simulated results were postprocessed with a MATLAB script for the FFT analysis.

For the experimental high-speed videos, the ripple wavelength (λ_R_), defined as the distance between two reverse peaks, was measured using NIH ImageJ, as previously described (49). Briefly, videos were converted to stacks, and λ_R_ was measured with the “plot profile” function. For the simulated data, λ_R_ was computed by postprocessing the biofilm surface contours using MATLAB. The ripple velocity (u_R_, the distance the wave travels in a given time) was calculated as u_R_ = f × λ_R_ = λ_R_/T.

### Numerical model.

The general assumptions made in the development of the numerical model were (i) the gas (air) and liquid (biofilm) phases are incompressible (Mach number below 0.3 for the gas phase); (ii) a uniform velocity profile, constant in time, leaves the compressor nozzle; (iii) the flow is symmetric with respect to the vertical axis (around the nozzle middle); (iv) the free gas jets are in the turbulent regime (due to calculated Re_a_); (v) the initial biofilm is a thin layer with constant thickness; and (vi) the biofilm is characterized by non-Newtonian fluid shear thinning (Herschel-Bulkley model) and density equal to that of water.

### Model geometry.

**FIG 9:**
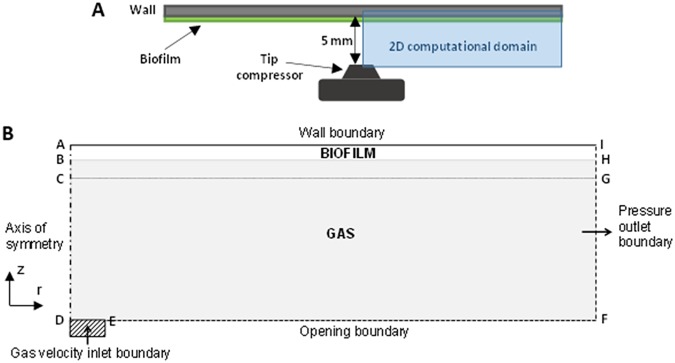
Experimental setup (A) and two-dimensional axisymmetric model (B) with radial (r) and axial (z) directions and the boundary conditions. A no-slip and zero-turbulence wall was imposed on the boundary AI, representing the glass microscope slide, the substratum on which the biofilm was grown. The air inlet was established on DE, and a pressure outlet condition was set on the boundary FI. A symmetry axis was used along on the boundary AD, and the boundary EF was open to the atmosphere.

The schematic representation of the experimental setup and the computational domain used to analyze the air jet impingements over biofilm thin layer are shown in Fig. 9A and B, respectively. A 2D axisymmetric computational domain represented the lateral view of the jet impact over biofilm, slicing the actual experimental setup. The domain length and height were L_x_ = 15 mm and L_y_ = 5 mm, respectively, with the biofilm initial thickness *L_b_* = 0.055 mm.

### Governing equations.

Momentum conservation (Equation 1) is coupled with continuity (Equation 2), as follows:

(1) $$\rho \frac{\partial \text{u}}{\partial t}+\rho \left(\text{u}\times \nabla \right)\text{u}=-\nabla p+\nabla \left[\left(\eta +\eta_{T}\right)\left(\nabla \text{u}+\nabla {\text{u}}^{T}\right)\right]+{\text{F}}_{\mathrm{st}}$$

(2) ρ∇×u=0

solved for the local velocity vector **u** and pressure *p*. **F***_st_* is the force arising from surface tension effects. The fluid density *ρ* and dynamic viscosity *η* were calculated by the VOF method in each control volume. *η_T_* is the turbulent viscosity, resulting from the k-ω turbulence model (see “Turbulence model” below). The interface between fluids (i.e., air and biofilm) was tracked with a robust coupling between level set and VOF methods, as implemented in the ANSYS Fluent software (51).

### Turbulence model.

An examination of Reynolds-averaged Navier-Stokes (RANS) modeling techniques recommends the shear-stress transport (SST) k-ω model instead of the standard k-ε model since it can better describe fluid flow in impinging jets within reasonable computational effort (50). The SST k-ω model incorporates a blending function to trigger the standard k-ω model in near-wall regions and the k-ε model in regions away from the wall.

The turbulence kinetic energy, k, and the specific dissipation rate, ω, are obtained from the transport equations including the convection and viscous terms, together with terms for production and dissipation of k and ω and cross-diffusion of ω. A user-defined source term for ω, representing the turbulence damping correction, was added to correctly model the flows in the interfacial area. Turbulence damping was needed because otherwise, the large difference in physical properties of biofilm and air phases would create a large velocity gradient at the interface, resulting in unrealistically high turbulence generation (52). See section A in File S1 in the supplemental material for more details.

### Biofilm viscosity.

The Herschel-Bulkley model (34) was used to characterize the dynamic viscosity of S. mutans biofilms, representing previously observed shear-thinning non-Newtonian behavior. The dynamic viscosity *η* (Pa·s) is inversely related to the shear rate $\dot{\gamma }$ (per second) and proportional to the shear stress *σ* (in pascals) as shown in Equation 3:

(3) $$\eta =\frac{\sigma }{\overset{˙}{\gamma }}$$

while the shear stress depends on the shear rate, shown in Equation 4:

(4) $$\sigma =\sigma_{y}+K\times {\overset{˙}{\gamma }}^{n}$$

with *σ_y_* representing the yield stress (in pascals), *K* representing the consistency index (Pa·s), and *n* representing the flow behavior index.

Since the calculated shear rates from CFD were so high that experimental data could not be obtained (orders of magnitude higher than obtainable with ordinary rheometers), we extrapolated using data from dynamic viscosity sweeps to determine the complex viscosity of S. mutans biofilms (28) and the dynamic viscosity of heterotrophic biofilms determined in reference 31, by modifying Herschel-Bulkley model parameters and evaluating numerically several viscosity curves from Equation 3. See the Results for more details.

### Boundary and initial conditions.

In the computational domain (Fig. 9B), a symmetr*y* axis was used on the boundary AD, with radial velocity component and normal gradients equal to zero. The boundary EF was open to the atmosphere, depending on the mass balance. A zero gauge pressure outlet condition was set on FI. The air inlet was on DE (half-nozzle size), with a fully developed velocity profile. The inlet turbulent energy, *k*, was computed as shown in Equation 5:

(5) $$k=\frac{3}{2}{\left(UI\right)}^{2}$$

with *U* indicating the mean flow velocity and *I* the turbulence intensity defined as *I* = 0.16 × *Re*^−1/8^ (23), and Reynolds number *Re* defined with velocity *U* and nozzle diameter. The specific turbulent dissipation rate, *ω*, was calculated as shown in Equation 6:

(6) $$\text{ω}={C_{\mu }}^{-1/4}\frac{\sqrt{k}}{l}$$

with the empirical constant *C_μ_* = 0.09 and *l* the turbulent length scale, assuming 7% of the nozzle diameter.

A no-slip and zero-turbulence wall was imposed on the biofilm substratum (boundary AI), with near-wall formulation to represent precisely the wall-bounded turbulent flow in the region, including the buffer layer and viscous sublayer. The *y*+-insensitive near-wall treatment (51) was used here, where based on the dimensionless wall distance of the first grid cell (*y*^+^), the linear and logarithmic law-of-the-wall formulations were blended. To resolve the viscous sublayer, the first grid cell needed to be at about *y*^+^ ≈ 1, also near the free surface (52). In the two-phase flow, the biofilm phase was initialized as a thin layer with constant thickness over the substratum, being several orders of magnitude more viscous than the air. The biofilm behaved initially like a solid, requiring resolving the viscous sublayer from the air-biofilm interface instead of from the wall as usually done.

In the initialization step, the values for *k* and *ω* were computed using Equations 5 and 6, with velocity *U* equal to the inlet velocity (*u_j_*) and characteristic length *l* = 1 mm, and the volume fraction of the biofilm phase was set to 1 in the region ABHI (Fig. 9B).

### Model solution.

**(i) Meshing.** A uniform mesh of prism cells was defined in the domain, with maximum size h_x_ by h_y_ of 50 μm by 17 μm, with a refined mesh in the region ACGI (minimum size, 15 μm by 0.4 μm) to satisfy the requirement *y*^+^ ≈ 1 near walls and in the free surface. A mesh growth rate no higher than 1.2 (51) was used between the refined subdomain and the remaining computational domain, leading to ∼450,000 mesh cells. Mesh details are shown in Fig. S1.

10.1128/mBio.02813-19.2 FIG S1 Details of the defined mesh in the computational domain. A refined mesh was defined in the region ACGI satisfying the requirement *y*^+^ ≈ 1. A mesh growth rate no higher than ≈1.2 was used from the refined region to mesh the remaining domain. Mesh quality was checked with the orthogonal quality parameter, which had average values of 1 in all domain, confirming the good quality of the defined regular mesh. Download FIG S1, TIF file, 0.2 MB. Copyright © 2020 Prades et al. 2020 Prades et al. This content is distributed under the terms of the Creative Commons Attribution 4.0 International license.

**(ii) Solvers.** The mathematical model was implemented into the commercial fluid dynamics software ANSYS Fluent (Academic Research, release 17.2). The governing equations were discretized using a second-order upwind scheme in space and first-order implicit in time, with pressure staggering option interpolation (PRESTO) and pressure-implicit splitting of operators (PISO) for the pressure-velocity coupling. The free surface deformation was tracked with the georeconstructed scheme. Transient simulations ran with a maximum time step set to 10^−7^ s for stable transient solutions. A total time of 20 ms was simulated in each run to reach a quasistationary solution.

**(iii) Simulation plan.** Two sets of simulations were carried out with the two-phase model. The first set (runs 1 to 7) was performed for model calibration, where the biofilm viscous properties were evaluated according to the experimental data. Experimental parameters, such as the measured jet velocity (*u_j_*) and biofilm thickness (*L_b_*), were used in this set with different non-Newtonian viscosities (*η*) (i.e., estimated viscosity curves [EVC]) and two surface tensions (*γ*). The second set (runs 8 to 12) was performed for sensitivity analysis, evaluating the effects of the inlet jet velocity and the biofilm thickness on the biofilm rippling response. Table 3 shows an overview of the numerical simulations.

**TABLE 3:** Overview of numerical simulations parameters

Data for run^*a*^	Run:											
	1	2	3	4	5	6	7	8	9	10	11	12
*η* (Pa·s)	EVC_3_	EVC_3_	EVC_1_	EVC_1_	EVC_2_	EVC_2_	EVC_4_	EVC_3_	EVC_3_	EVC_3_	EVC_3_	EVC_3_
*γ* (mN·m^-1^)	36	72	36	72	36	72	36	36	72	36	72	36
*L_b_* (μm)	55	55	55	55	55	55	55	55	55	55	55	27.5
*u_j_* (m·s^-1^)	41.7	41.7	41.7	41.7	41.7	41.7	41.7	20	20	60	60	41.7

a *η*, biofilm viscosity; *γ*, surface tension; *L_b_*, biofilm thickness; *u_j_*, jet velocity.

10.1128/mBio.02813-19.1 FILE S1 Computational and experimental investigation of biofilm disruption dynamics induced by high-velocity gas jet impingement. Download Text S1, DOCX file, 0.1 MB. Copyright © 2020 Prades et al. 2020 Prades et al. This content is distributed under the terms of the Creative Commons Attribution 4.0 International license.
