# Supplementary figures and images for: Computational and Experimental Investigation of Biofilm Disruption Dynamics Induced by High-Velocity Gas Jet Impingement
Source: mBio. 2020 Jan 7;11(1):e02813-19. doi: 10.1128/mBio.02813-19 (PMC6946800; doi:10.1128/mBio.02813-19)

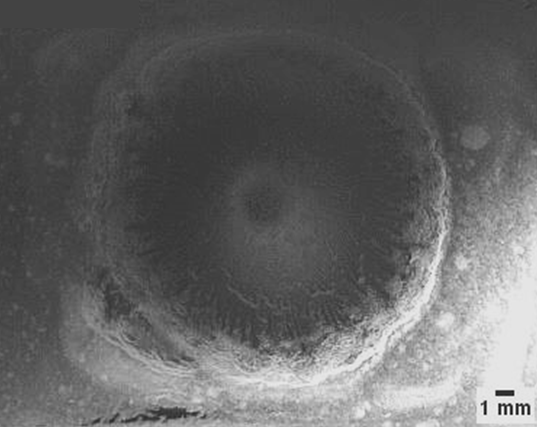

Supplement: FIG S2 [file mBio.02813-19-sf002.tif]

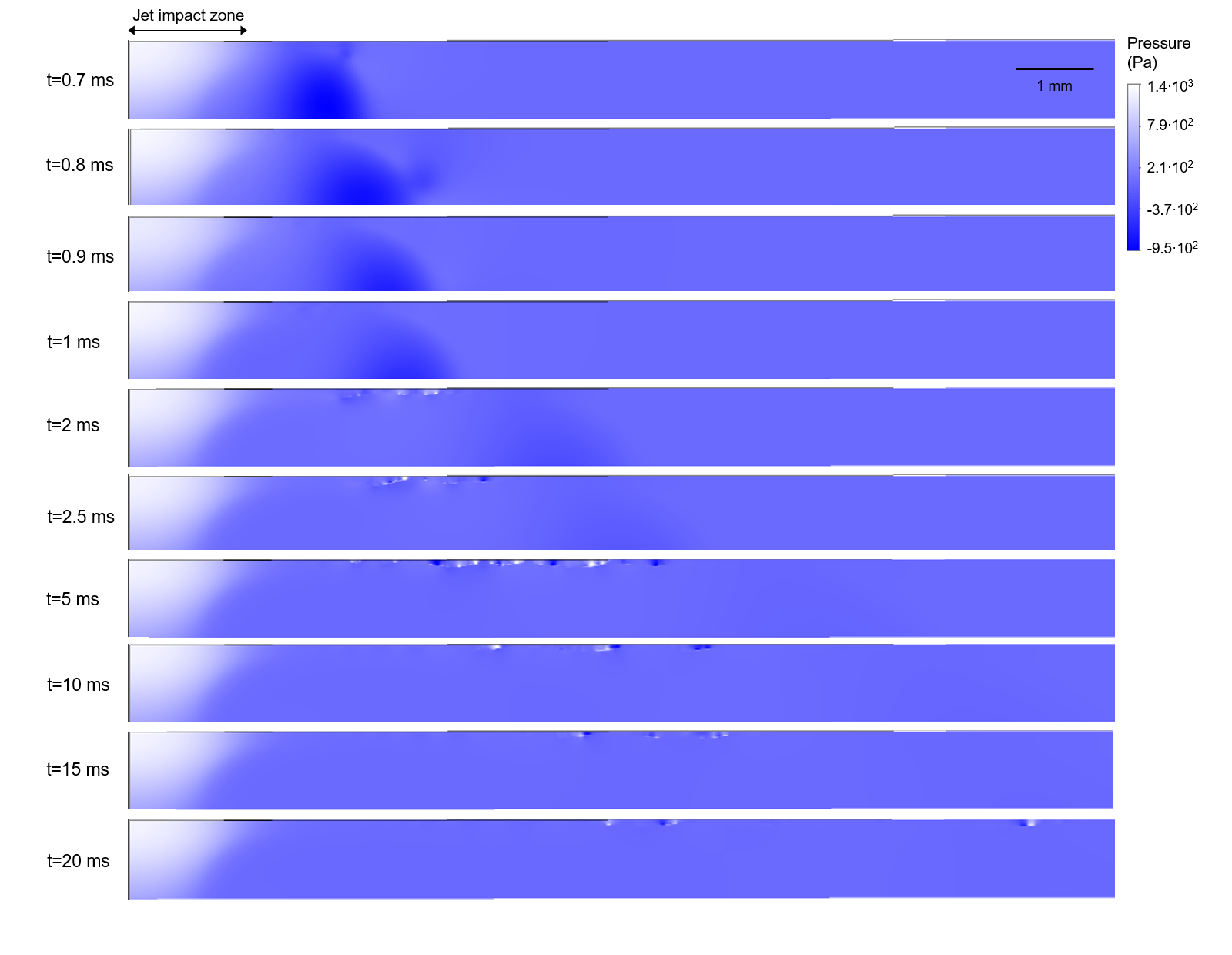

Supplement: FIG S3 [file mBio.02813-19-sf003.tif]

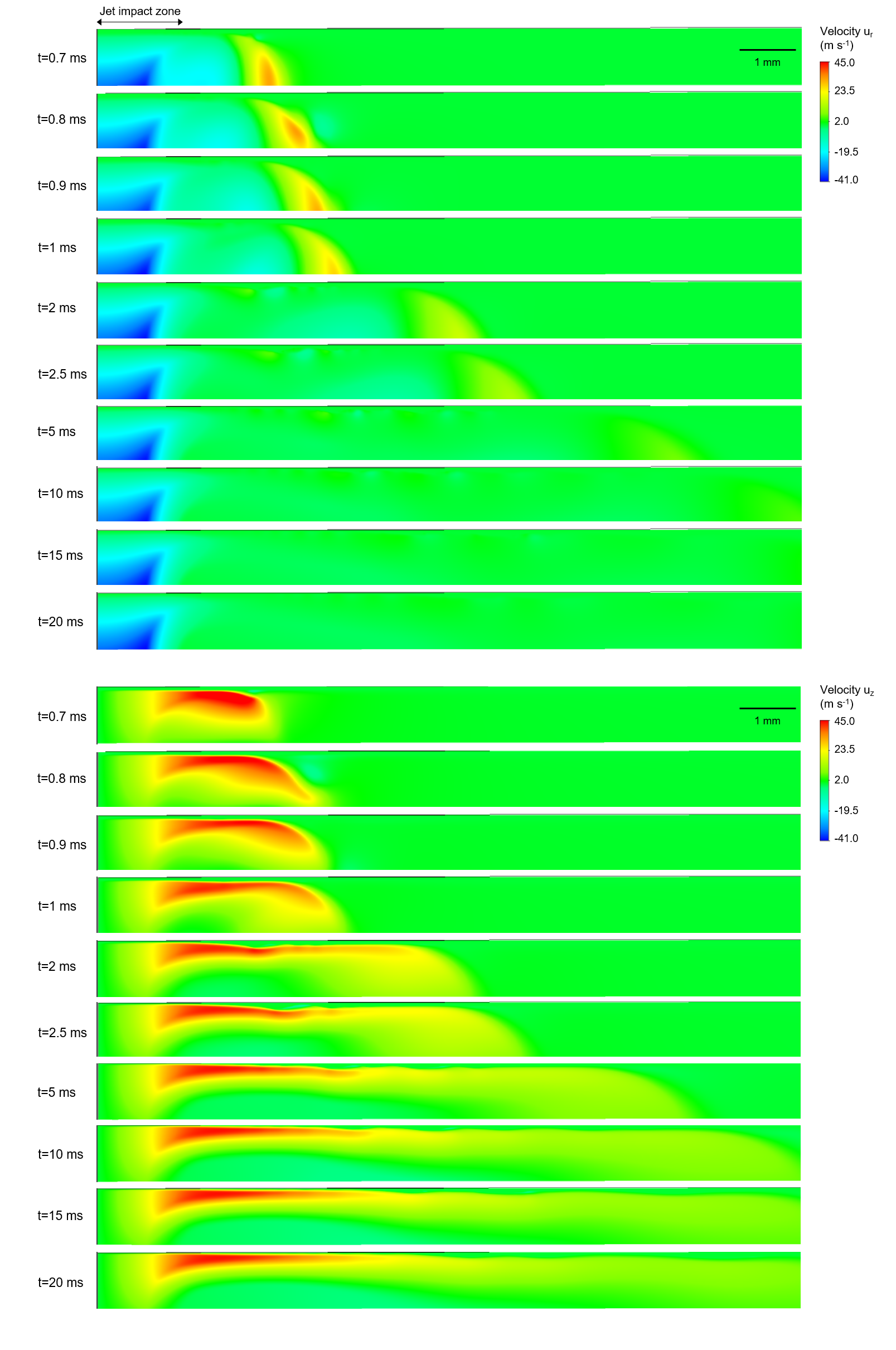

Supplement: FIG S4 [file mBio.02813-19-sf004.tif]

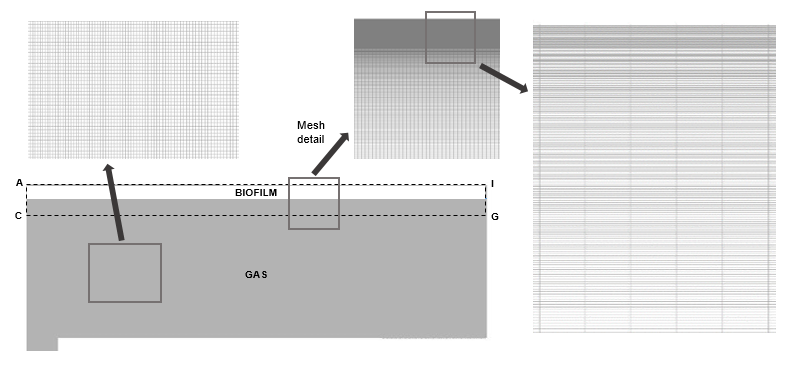

Supplement: FIG S1 [file mBio.02813-19-sf001.tif]
