# Supplementary material for: Computational and Experimental Investigation of Biofilm Disruption Dynamics Induced by High-Velocity Gas Jet Impingement
Source: mBio. 2020 Jan 7;11(1):e02813-19. doi: 10.1128/mBio.02813-19 (PMC6946800; doi:10.1128/mBio.02813-19)
Supplement: FILE S1 [file mBio.02813-19-s0001.docx]

**SUPPLEMENTARY INFORMATION**

**Computational and experimental investigation of biofilm disruption dynamics induced by high velocity gas jet impingement**

Lledó Prades^a^, Stefania Fabbri^b^, Antonio D. Dorado^a^, Xavier Gamisans^a^, Paul Stoodley^c,d^#, Cristian Picioreanu^e^

^a^ Department of Mining, Industrial and ICT Engineering, Universitat Politècnica de Catalunya, Manresa, Spain

^b^ Perfectus Biomed Limited, Sci‐Tech Daresbury, Cheshire, UK

^c^ Department of Microbial Infection and Immunity and Department of Orthopaedics, The Ohio State University, Columbus, Ohio, USA

^d^ National Centre for Advanced Tribology at Southampton (nCATS), Department of Mechanical Engineering, University of Southampton, Southampton, UK;

^e^ Department of Biotechnology, Delft University of Technology, Delft, The Netherlands

#Address correspondence to Paul Stoodley, [Paul.Stoodley@osumc.edu](mailto:Paul.Stoodley@osumc.edu)

1. **Turbulence model equations**

The turbulence kinetic energy, *k*, and the specific dissipation rate, *ω*, are obtained from the following transport equations, respectively:

$\rho\frac{\partial k}{\partial t}+\rho\left(\mathbf{u}\cdot\nabla\right)k=\nabla\cdot\left[\left(\Gamma_{k}\right)\nabla k\right]+G_{k}-Y_{k}+S_{k}$ (S.1)

$\rho\frac{\partial\omega}{\partial t}+\rho\left(\mathbf{u}\cdot\nabla\right)\omega=\nabla\cdot\left[\left(\Gamma_{\omega}\right)\nabla\omega\right]+G_{\omega}-Y_{\omega}+D_{\omega}+S_{\omega}$ (S.2)

where *Γ_k_* and *Γ_ω_* represent the effective diffusivity of *k* and *ω*, respectively. *G_k_* represents the production of *k,* and *G_ω_* represents the generation of *ω*. *Y_k_* and *Y_ω_* represent the dissipation of *k* and *ω* due to turbulence. *D_ω_* represents the cross-diffusion term. *S_k_* and *S_ω_* are user-defined source terms for *k* and *ω*, respectively. More details about this model and definition of the individual terms can be found in (1).

**A.1. Turbulence modeling in the interface**

When a multiphase system is considered, the physical properties of the phases present in the model should be taken into account, since an enormous contrast among them can be detected across the interface. This difference is manifested as a high-velocity gradient at the interface between two fluids, resulting in high turbulence generation in both phases. In this case, certain damping of turbulence is required for the accurate modeling of the interfacial area and the fluids flow behavior.

Various works (2–4) have validated the use of the turbulence damping correction model. In ANSYS Fluent, the turbulence damping is available for the k-ω models. As described by Egorov et al. (2), the turbulence damping adds the following term *S_i_* (eq. S.3) as a source *S_ω_* to the ω-equation (eq. S.2):

$S_{i}=A_{i}\Delta n\beta\rho_{i}\left(\frac{B6\eta_{i}}{\beta\rho_{i}\Delta n^{2}}\right)^{2}$ (S.3)

where *A_i_* is the interfacial area density for the phase i, $\Delta n$ is the cell height normal to the interface, *β* is the k-ω model closure coefficient (*β*=0.075), *ρ_i_* is the density of phase i, *B* is the damping factor and *η_i_* is the viscosity of phase i. The factor *A_i_* activates the source term *S_ω_* only at the free surface, where it cancels the standard ω destruction term of the ω-equation (*Y_ω_*) and enforces the required high value of *ω*, and thus the turbulence damping.

The $\Delta n$ is calculated internally by the software using grid information, and a damping factor *B*=10 was used in this work, since bigger values do not change the result for a solid wall (2). In addition, the interfacial area density is computed as a function of the volume fraction:

$A_{i}=2\alpha_{i}\left|\nabla\alpha_{i}\right|$ (S.4)

where *α_i_* is the volume fraction of phase i.

Additionally, the turbulence damping model near a free surface requires a mesh which resolves the viscous sublayer near a free surface, i.e. the grid should have y+ ≈ 1 near the interface (2).

1. **Meshing and experimental and simulated results**

Details regarding the defined computational mesh, the experimental analysis on air-jet impingement over biofilms, and the simulated results reproducing biofilm disruption are shown in this section by means of figures and videos. The different figures and videos are listed below.

**LIST OF FIGURES**

**Figure S1.** Details of the defined mesh in the computational domain. A refined mesh was defined in the region ACGI satisfying the requirement y^+^≈1. A mesh growth rate no higher than ≈1.2 was used from the refined region to mesh the remaining domain. Mesh quality was checked with the orthogonal quality parameter, which had average values of 1 in all domain, confirming the good quality of the defined regular mesh.

**Figure S2.** Steady-state of the biofilm disruption after perpendicular air-jet impingement. The ripples die out when the biofilm flowed to the cleared space edge after ~350 ms of jet exposure.

**Figure S3.** Computed pressure fields in the disrupted region (rectangle marked in Figure 6) for different times, i.e. 0.7, 0.8, 0.9, 1, 2, 2.5, 5, 10, 15, and 20 ms. Simulations were performed with *η*=EVC_3_ and *γ*=36 mN·m^-1^. Color scale: pressure in Pa. Larger pressures were in the air-jet impact zone. Pressure gradients formed in the in the biofilm phase were observed from t=2 ms.

**Figure S4.** Computed air-jet velocity profiles in X and Y directions (velocity u_r_ and u_z_, respectively) in the disrupted region (see Figure 6) for different times, i.e. 0.7, 0.8, 0.9, 1, 2, 2.5, 5, 10, 15, and 20 ms. Simulations were performed with *η*=EVC_3_ and *γ*=36 mN·m^-1^. Color scale: Velocity in m·s^-1^. Larger velocities in the gas phase were produced in the tangential direction to the biofilm phase, reaching maximum values of 45 m·s^-1^.

**LIST OF VIDEOS**

**Video S1.** High-speed (2000 fps) recording of the air jet impingement experiment on a *S. mutans* biofilm attached to a glass surface, at an air velocity of 41.7 m·s^-1^, with a nozzle diameter of 2 mm. The biofilm consisted or larger clusters (lighter patches) distributed heterogeneously and separated by a base biofilm (grey). In the first few frames the shutter can be seen being lifted out of the way exposing the biofilm to the air jet. Biofilm ripples are forming radially away from the central impact area. After approximately 200 ms the biofilm had been “pushed” from the central area to from a cleared area (darker) of approximately 1.5 cm. This video was used to generate data for the computational analysis.

**Video S2.** Simulated biofilm ripples formation in time (X-Y top view), from a biofilm thickness of 55 μm with viscosity model EVC3 and surface tension γ=36 mN·m^-1^, subjected to a perpendicular air jet with velocity of 41.7 m·s^-1^.

**Video S3.** Simulated changes of biofilm thickness in time (0 – 20 ms) over the radial direction (X-Z lateral view) for viscosity model parameters EVC_3_ and surface tension *γ*=36 mN·m^-1^.

**Video S4.** Simulated biofilm ripples formation over time (X-Z lateral view), from a biofilm thickness of 55 μm with viscosity model EVC_3_ and surface tension *γ*=36 mN·m^-1^, subjected to a perpendicular air jet with velocity of 41.7 m·s^-1^. Colored surface: velocity magnitude (m·s^1^); Gray surface: biofilm area. In this simulation the jet is coming from the bottom of the screen and the biofilm is on the top.

**Video S5.** Simulated biofilm ripples formation over time (X-Z lateral view), from a biofilm thickness of 55 μm with viscosity model parameters EVC_3_ and surface tension *γ*=36 mN·m^-1^, subjected to a perpendicular air jet with velocity of 41.7 m·s^-1^. Gray-scale surface: shear rate (s^-1^); Color-scale: biofilm dynamic viscosity (Pa⋅s). In this simulation the jet is coming from the bottom of the screen and the biofilm is on the top.

**REFERENCES**

1. ANSYS Inc. 2016. ANSYS® Academic Res//earch, Release 17.2, Help System, ANSYS Fluent Theory Guide.

2. Egorov Y, Boucker M, Martin A, Pigny S, Scheuerer M, Willemsen S. 2004. Validation of CFD codes with PTS-relevant test cases, p. 91–116. *In* 5th Euratom Framework Programme ECORA project, CONTRACT No FIKS-CT-2001-00154.

3. Vallée C, Höhne T, Prasser HM, Sühnel T. 2008. Experimental investigation and CFD simulation of horizontal stratified two-phase flow phenomena. Nucl Eng Des 238:637–646.

4. Höhne T, Mehlhoop JP. 2014. Validation of closure models for interfacial drag and turbulence in numerical simulations of horizontal stratified gas-liquid flows. Int J Multiph Flow 62:1–16.
